# Supplementary material for: imGLAD: accurate detection and quantification of target organisms in metagenomes
Source: PeerJ. 2018 Nov 2;6:e5882. doi: 10.7717/peerj.5882 (PMC6216955; doi:10.7717/peerj.5882)
Supplement: Supplemental Information 1 — Supplementary Figures S1–S8. [file peerj-06-5882-s001.docx]

**SUPPLEMENTARY FIGURES AND TABLES**

**imGLAD: accurate detection and quantification of target organisms in metagenomes**

Juan C. Castro, Luis M. Rodriguez-R, Michael R. Weigand, Janet K. Hatt, Michell Q. Carter, and Konstantinos T. Konstantinidis

**Supplementary Figure 1.**

**
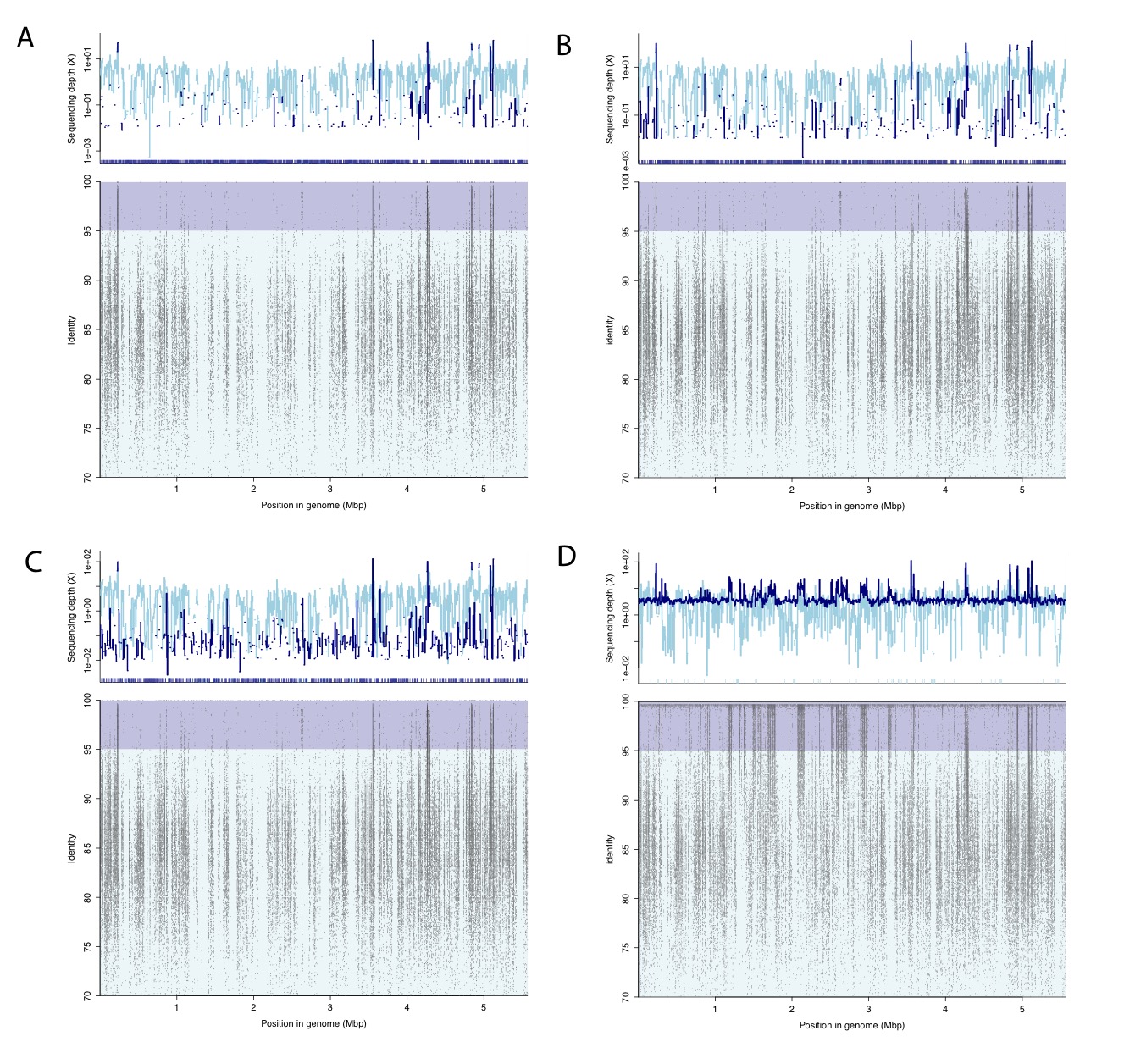
**

**Supplementary Figure 1. Read recruitment plots for the *E. coli* inoculation experiment**. Each dot represents a read mapped (recruited) against the reference *E. coli* str. O157:H7 EC11 genome; X axes show the position in the genome in which the read was aligned to and Y axes show the identity of the corresponding alignment. Panels show the different experiments: A. Control (no *E. coli* cell inoculated), B. 80, C. 8000, and **D**. 800000 inoculated cells. Upper panel represents the sequencing depth provided by the reads mapped on the genome at species level (i.e., >95% nucleotide identity; Dark blue) and below species level (Cyan).

**Supplementary Figure 2.**

**
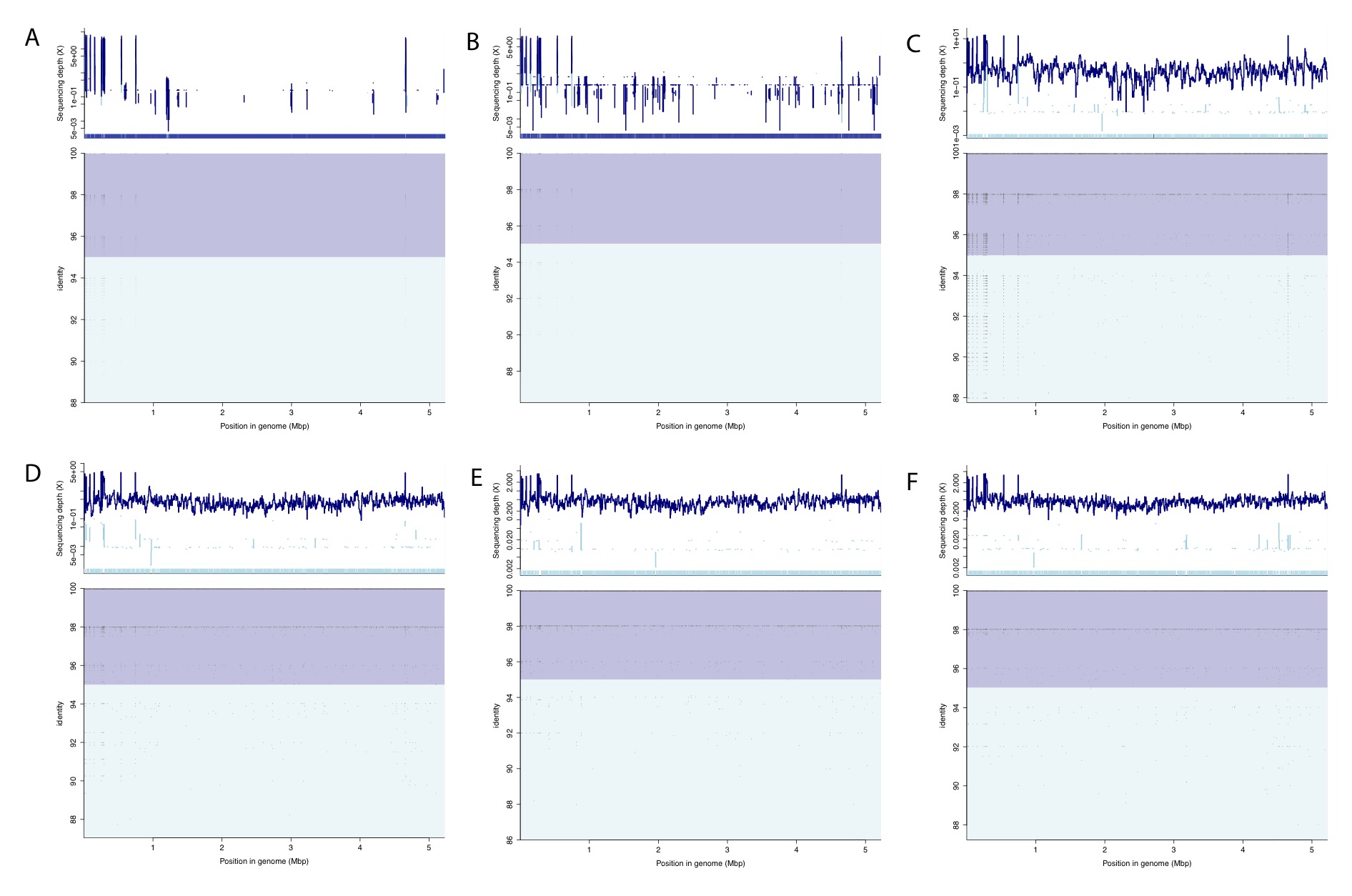
**

**Supplementary Figure 2. Read recruitment plots for the publicly available *B. anthracis* datasets**. Each dot represents a read mapped (recruited) against the reference *B. anthracis* str. ames genome; X axes show the position in the genome in which the read was aligned to and Y axes show the identity of the corresponding alignment. Panels show the different experiments: A. DNA of 1 Genome was added, B. 10, C. 100, **D.** 1000, **E.** 10000 and, **F.** 100000 added genomes. Upper panel represents the sequencing depth provided by the reads mapped on the genome at species level (i.e., >95% nucleotide identity; Dark blue) and below species level (Cyan).

**Supplementary Figure 3.**

**Supplementary Figure 3. Assessment of tool accuracy based on 571 datasets of the HMP project spiked with different concentrations of reads from *E. coli* and close relatives**. This figure is similar to Figure 3C of the main manuscript except that 571 HMP metagenomes were used to provide for a naturally occurring background community (as opposed to *in-silico* generated datasets) and sensitivity and specificity percentages were combined as accuracy (harmonic mean of sensitivity and specificity). The metagenomes were spiked with 10 *E. coli* genomes with ANI ranging between 95-98% to the target *E. coli* O157:H7 EC11 genome at the same concentration (i.e., 0.3X), in addition to varied levels of the target *E. coli* O157:H7 EC11 reads (x-axis). Note that as sequencing breadth increases the accuracy of the prediction is higher for all tools tested, and imGLAD performs as well as, or better (i.e., ~100% accuracy level) than alternative tools.

**Supplementary Figure 4.**

**
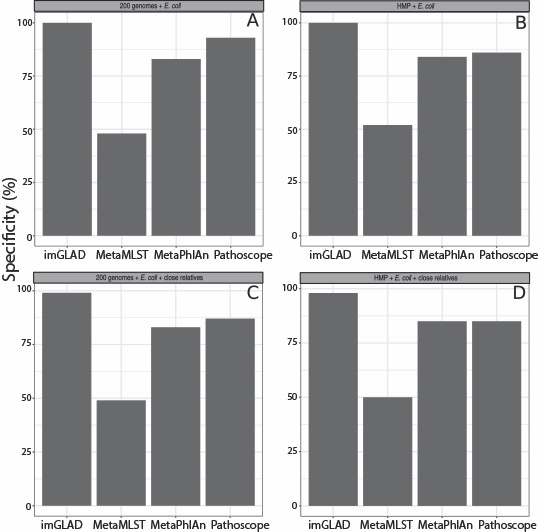
**

**Supplementary Figure 4. Specificity of imGLAD compared to other tools**. The specificity, i.e., the fraction of correctly identified negative datasets among all negative datasets examined, of the tools was determined for different datasets. **A**. *in-silico* synthesized negative datasets from 200 RefSeq genomes excluding *E. coli*. **B**. Similar comparisons based on 571 datasets from the HMP project, which did not contain any spiked *E. coli* reads. **C**. imGLAD was evaluated in the same datasets as in panel A but this time the datasets included, in addition to the RefSeq genomes, 10 *E. coli* genomes with ANI ranging between 95-98% to the target *E. coli* O157:H7 EC11 genome spiked in at several different concentrations. **D**. 571 datasets from the HMP project were spiked with 10 evenly sampled *E. coli* genomes (i.e., 0.3X)*,* whereas *E. coli* O157-H7 was not included (absent). Note that imGLAD’s specificity is higher than any other tool tested for all datasets.

**Supplementary Figure 5.**

**
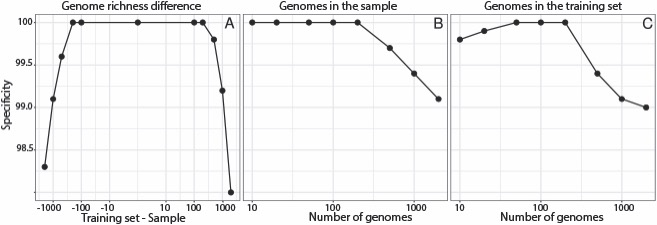
**

**Supplementary Figure 5. Effect of the number of genomes used in the training dataset and query sample on imGLAD’s specificity**. Specificity, i.e., the fraction of correctly identified negative datasets among all negative datasets examined, was assessed for several key parameters of the imGLAD pipeline based on *in-silico* synthesized query datasets (samples). Target *E. coli* was spiked at 0.03 sequencing breadth for all the query samples. **A**. Difference in genome richness between training datasets and query sample caused a decrease in specificity when the difference was larger than about 2 orders of magnitude. **B**. Training datasets based on 200 genomes were tested against query samples with variable richness, and specificity decreased when the difference between training set and sample increased. **C**. *in-silico* synthesized query samples that included 200 genomes were tested with training datasets of different number of genomes, and specificity decreased with training datasets based on a small number of genomes as well as with a much higher number of genomes than those in the sample (e.g., higher than 2 orders of magnitude). Note that in all cases, the decrease in specificity was rather minor, i.e., 1-3%, and training datasets with 200 genomes should give reliable results for most natural communities of low to high complexity.

**Supplementary Figure 6.**

**
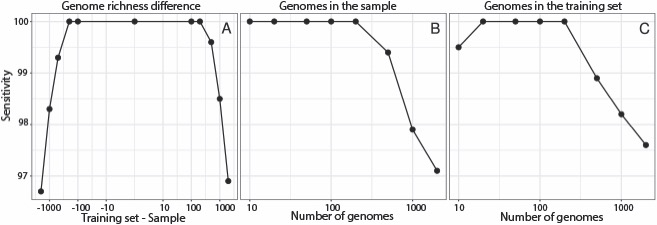
**

**Supplementary Figure 6. Effect of number of genomes used in the training datasets and query sample on imGLAD’s sensitivity**. The figure is identical to Figure 6 above except that sensitivity is shown, i.e., the proportion of properly classified positive datasets among the total number of positive datasets.

**Supplementary Figure 7.**

**Supplementary Figure 7. Effects of the dataset (library) size on the limit of detection of imGLAD**. At small library sizes the percentage of the genome covered by negative reads (i.e. spurious matches and highly conserved or horizontally transferred regions) is small due to fewer reads available, which yields a (inaccurate) low detection limit of detection for sequencing breadth (y-axis). However, as the library size increase, the regions recruiting negative reads become saturated and the detection limit stabilizes at about 100,000 reads, which is the default dataset size used in imGLAD for training datasets.

**Supplementary Figure 8.**

**Supplementary figure 8.** Receiver operating characteristic of imGLAD and Pathoscope. Because separation of positive and negative samples is almost perfect, the logistic function is able to separate both sets efficiently, which yields low number of false negatives at low values of sequencing breadth and low number of false positives at higher values. Exceptions to this only occur in cases where highly related, non-target close relatives (e.g., showing >98.5% ANI to the target) are present in the training dataset. The specific example shown here is from *E. coli* with 98% non-target relatives being present.
